# Reconstruction of shoulder MRI using deep learning and compressed sensing: a validation study on healthy volunteers

**DOI:** 10.1186/s41747-023-00377-2

**Published:** 2023-10-26

**Authors:** Thomas Dratsch, Florian Siedek, Charlotte Zäske, Kristina Sonnabend, Philip Rauen, Robert Terzis, Robert Hahnfeldt, David Maintz, Thorsten Persigehl, Grischa Bratke, Andra Iuga

**Affiliations:** 1 Department of Diagnostic and Interventional Radiology, University of Cologne, Faculty of Medicine and University Hospital Cologne, Kerpener Str. 62, 50937 Cologne, Germany; 2 Philips GmbH Market DACH, Hamburg, Röntgenstrasse 22, 22335 Hamburg, Germany

**Keywords:** Artifacts, Artificial intelligence, Deep learning, Magnetic resonance imaging, Shoulder joint

## Abstract

**Background:**

To investigate the potential of combining compressed sensing (CS) and deep learning (DL) for accelerated two-dimensional (2D) and three-dimensional (3D) magnetic resonance imaging (MRI) of the shoulder.

**Methods:**

Twenty healthy volunteers were examined using at 3-T scanner with a fat-saturated, coronal, 2D proton density-weighted sequence with four acceleration levels (2.3, 4, 6, and 8) and a 3D sequence with three acceleration levels (8, 10, and 13), all accelerated with CS and reconstructed using the conventional algorithm and a new DL-based algorithm (CS-AI). Subjective image quality was evaluated by two blinded readers using 6 criteria on a 5-point Likert scale (overall impression, artifacts, and delineation of the subscapularis tendon, bone, acromioclavicular joint, and glenoid labrum). Objective image quality was measured by calculating signal-to-noise-ratio, contrast-to-noise-ratio, and a structural similarity index measure. All reconstructions were compared to the clinical standard (CS 2D acceleration factor 2.3; CS 3D acceleration factor 8). Additionally, subjective and objective image quality were compared between CS and CS-AI with the same acceleration levels.

**Results:**

Both 2D and 3D sequences reconstructed with CS-AI achieved on average significantly better subjective and objective image quality compared to sequences reconstructed with CS with the same acceleration factor (*p* ≤ 0.011). Comparing CS-AI to the reference sequences showed that 4-fold acceleration for 2D sequences and 13-fold acceleration for 3D sequences without significant loss of quality (*p* ≥ 0.058).

**Conclusions:**

For MRI of the shoulder at 3 T, a DL-based algorithm allowed additional acceleration of acquisition times compared to the conventional approach.

**Relevance statement:**

The combination of deep-learning and compressed sensing hold the potential for further scan time reduction in 2D and 3D imaging of the shoulder while providing overall better objective and subjective image quality compared to the conventional approach.

**Trial registration:**

DRKS00024156.

**Key points:**

• Combination of compressed sensing and deep learning improved image quality and allows for significant acceleration of shoulder MRI.

• Deep learning-based algorithm achieved better subjective and objective image quality than conventional compressed sensing.

• For shoulder MRI at 3 T, 40% faster image acquisition for 2D sequences and 38% faster image acquisition for 3D sequences may be possible.

**Graphical Abstract:**

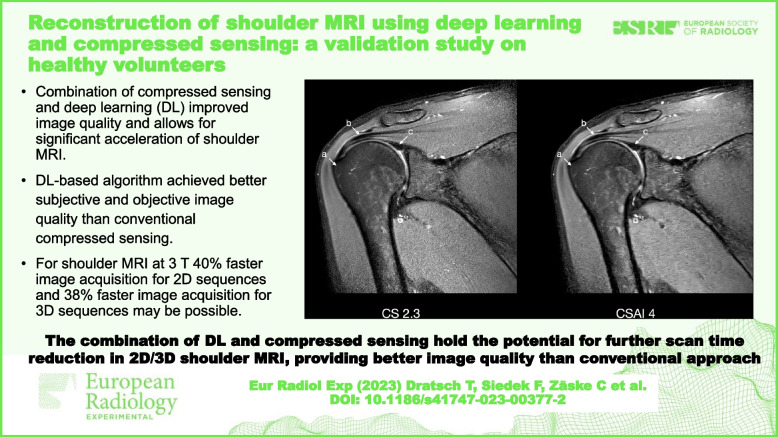

## Background

The shoulder is the third most common site of musculoskeletal pain [1]. Common injuries of the shoulder, such as rotator cuff tears, have a high prevalence in the general population [2, 3], negatively affect productivity and quality of life [4], and are associated with a high socioeconomic burden [5]. Magnetic resonance imaging (MRI), with its high tissue contrast, plays a critical role in the diagnosis and treatment evaluation of shoulder injuries [6]. However, with an ever increasing demand for MRI [7] and acquisition times for an MRI of the shoulder ranging between 15 and 20 min [8], the number of patients that can be imaged at a given time is limited. Thus, several possibilities, such as reduced scanning protocols [8], or new techniques such as parallel imaging (*e.g.*, generalized autocalibrating partial parallel acquisition, GRAPPA) [9], and compressed sensing (CS) [10], have been developed to increase the speed of image acquisition without sacrificing image quality. The CS approach uses random undersampling of the k-space to reconstruct the MRI signal with fewer measurements, thereby reducing image acquisition times. Several studies have shown that CS can be used to reduce scan times for multiple anatomical regions [11–14], including the shoulder [15], while still maintaining diagnostic image quality. However, artifacts, such as aliasing and blurring, which are introduced at higher acceleration factors, limit further acceleration of image acquisition using conventional CS.

With the recent advent of deep learning (DL) and its integration into radiology, one approach that has been suggested to overcome those limitations is to combine CS and DL [16]. As part of the 2019 fastMRI challenge, Pezzotti et al. [16, 17] introduced Adaptive-CS-Net, a neural network for improved reconstruction of MRI of the knee, which allowed reduced acquisition times by a factor of 8. Adaptive-CS-Net was then further developed to extend to a wider range of acceleration factors and anatomical regions, including the shoulder. However, so far, there have been no studies testing the clinical feasibility and limitations of Adaptive-CS-Net to accelerate MR images of the shoulder. Thus, the aim of the current study was to determine whether combining CS with DL is a feasible approach to further decrease the acquisition times of shoulder MR images while maintaining diagnostic image quality.

## Methods

### Study population

This single-center study was approved by our institutional review board and registered in the national Clinical Trials Register (DRKS00024156). Recruitment of volunteers and acquisition of imaging data were carried out from February to March 2022. Written informed consent was obtained from all participants included in the study. Exclusion criteria were pregnancy, age below 18, implanted MRI conditional or unsafe devices, previous surgery or known pathologies of the shoulder, and shoulder related pain in the last 6 months.

### MRI acquisition and reconstruction

A whole-body 3-T MRI system (Philips Ingenia 3.0 T, Philips, Amsterdam, The Netherlands) with a dedicated receiver 8-channel shoulder coil was used. All volunteers were placed supine, head-first on the table. For all sequences, the field-of-view covered the entire shoulder joint. The protocol included a fat-saturated two-dimensional (2D) coronal proton density-weighted sequence with four different acceleration levels (2.3, 4, 6, and 8) as well as a fat-saturated three-dimensional (3D) coronal proton density sequence with three different acceleration levels (8, 10, and 13). Except for the acceleration factors, all other parameters were kept identical between the acquired sequences. Table 1 summarizes the sequence parameters used in this study. The sets of undersampled k-space data from the different acceleration levels were reconstructed into visually perceivable images using two methods: (1) a conventional approach (CS) and (2) a novel artificial intelligence (AI)-driven prototype (CS-AI).

**Table 1:** Acquisition parameters for the different sequences and results for changes in the scan time

	**Two-dimensional sequences**				**Three-dimensional sequences**		
**Sequence/Parameter**	CS 2.3 CS-AI 2.3	CS 4 CS-AI 4	CS 6 CS-AI 6	CS 8 CS-AI 8	CS 8 CS-AI 8	CS 10 CS-AI 10	CS 13 CS-AI 13
**Echo time [ms]**	40	40	40	40	152	152	152
**Repetition time [ms]**	4,169	4,169	3,917	3,917	1,100	1,100	1,100
**Flip angle [degrees]**	90	90	90	90	90	90	90
**Field of view [mm]**	160 × 160	160 × 160	160 × 160	160 × 160	160 × 177 × 100	160 × 177 × 100	160 × 177 × 100
**Slice thickness [mm]**	3	3	3	3	−	−	−
**Number of slices**	36	36	36	36	−	−	−
**Gap [mm]**	0.3	0.3	0.3	0.3	−	−	−
**Acquisition voxel size [mm]**	0.4 × 05.5	0.4 × 05.5	0.4 × 05.9	0.4 × 05.7	0.7 × 0.7 × 0.7	0.7 × 0.7 × 0.7	0.7 × 0.7 × 0.7
**Reconstruction voxel size [mm]**	0.2 × 0.2	0.2 × 0.2	0.2 × 0.2	0.2 × 0.2	0.34 × 0.34 × 0.4	0.34 × 0.34 × 0.4	0.34 × 0.34 × 0.4
**Turbo factor/Echo train length**	12	12	10	10	35	35	35
**CS factor**	2.3	4	6	8	8	10	13
**Scan time [s]**	167	100	78	63	289	232	179
**Saved scan time [s]**	−	67	89	104	−	57	110
**Scan time reduction [%]**	−	40	53	62	−	19	38

Only the acceleration factors were changed between the different sequences to keep them as comparable as possible

*CS* Compressed sensing*, CS-AI* Compressed sensing combined with a deep learning-based algorithm

The CS-AI reconstruction technique used in this study builds on compressed sensitivity encoding (SENSE), in which the parallel imaging technique SENSE and compressed sensing are integrated into a single acceleration technique. Compressed SENSE is based on a non-uniform pseudorandom sampling scheme with multiple receiver coil elements after which an iterative reconstruction scheme is performed in which a data consistency term and a sparsity constraining term are balanced. The required sparsity is given in the wavelet domain and data consistency is preserved while performing an iterative, regularized L1 minimization reconstruction technique. Being based on the compressed SENSE acquisition scheme, CS-AI employs a DL convolutional neural network (“Adaptive—CS-Net”) introduced by Pezzotti et al. [16, 17]. The reconstruction of the original CS sampled data is improved by replacing the iterative, regularized L1 minimization reconstruction scheme by a set of multiscale network blocks in which in each block, a data consistency check per coil element is performed. This is similar to the CS reconstruction and avoids deviation of the resulting image from the measured data, prevents the introduction of phantom structures, and minimizes data loss. Compared to the implementation by Pezzotti et al. [16, 17], where network training was exclusively performed on a large-scale dataset of knee data, the algorithm was extended by using training data of about 740,000 MRI images with various anatomies, contrasts, and field strengths (1.5 and 3 T). Both the acquisition and reconstruction algorithms (CS and CS-AI) were provided by the manufacturer (Compressed SENSE, Philips Healthcare).

### Subjective image analysis

All scans were exported as DICOM files to the clinical Picture Archiving and Communication System (Impax EE R20, Agfa Healthcare, Mortsel, Belgium). Two radiologists with 4 and 8 years of experience independently reviewed all images. For the subjective reading, the images were presented in random order and both readers were blinded to the scan sequence and reconstruction. All blinded images of a subject were available at once for both readers. Readers were free to choose window width and level settings, and the review was performed over a period of 6 weeks. Using a 5-point Likert scale, each reader independently evaluated the delineation of the following anatomical structures for all sequences: subscapularis tendon, bone, acromioclavicular joint, and glenoid labrum. Overall image impression and visible artifacts were rated additionally on a 5-point Likert scale, resulting in a total of 6 subjective ratings for each of the 14 images (2D: CS 2.3/CS-AI 2.3, CS 4/CS-AI 4, CS 6/CS-AI 6, CS 8/CS-AI 8; 3D: CS 8/CS-AI 8, CS 10/CS-AI 10, CS 13/CS-AI 13) reconstructed for every patient. Table 2 shows an overview of the used scale.

**Table 2:** Ratings for the anatomical structures, diagnostic certainty/overall image impression, and artifacts

**Level**	**Anatomical structures**	O**verall image impression**	**Artifacts**
**1**	Not visible/distinguishable	Not acceptable/ no diagnostic value	Massive artifacts
**2**	Barely visible	Very limited diagnostic value	Significant artifacts
**3**	Adequately visible	Acceptable for most diagnoses	Acceptable artifacts
**4**	Good visibility	Good for majority of diagnoses	Minimal artifacts
**5**	Excellent visibility	Optimal	No artifacts

### Objective image analysis

#### Objective image analysis: region of interest (ROI)-based

The Picture Archiving and Communication System was used for manual positioning of ROI in the following anatomical structures of the shoulder joint: muscle (deltoid muscle), bone (proximal humerus), and tendon (supraspinatus tendon). Signal pathology in the respective areas was excluded prior to measurement. Average ROI were 92.10 ± 1.37 mm^2^ (mean ± standard deviation [SD]) for the bone, 91.70 ± 2.10 mm^2^ for the muscle, and 5.85 ± 1.68 mm^2^ for the tendon measurements. Similar to Lee et al. [18], signal-to-noise ratios (SNRs) for the bone, muscle, and tendon were derived from the ROIs by dividing the average signal intensity (SI) value by the standard deviation of the tissue. Additionally, contrast-to-noise ratios (CNRs) for the bone-tendon, tendon–muscle, and bone-muscle were calculated with the following equations, as described in previous studies [12, 18, 19]:

$$\left|(SI\ a-SI\ b)/\sqrt{\left({SD\ a}^{2}+{SD\ b}^{2}\right)}\right|.$$

#### Objective image analysis: pixel-based

To assess the similarity between the accelerated images and the reference sequences, we also calculated the structural similarity index measure (SSIM) using CS 2.3 for 2D sequences and CS 8 for 3D sequences as the reference [20]. An in-house tool developed using the scikit-image toolbox was used to carry out a pixel-wise analysis of the central slice of each scan [21–23]. The resulting SSIM values represent a percentual deviation for each sequence from the reference scan, with higher values indicating greater similarity to the reference image.

### Statistical analysis

GraphPad Prism version 9.0.1 for Mac OS X (GraphPad Software, Boston, USA) was used for all statistical analyses. For the subjective image analysis, the values from both readers for each sequence were averaged. To assess the interrater agreement between both readers, Krippendorff’s alpha was calculated, with a Krippendorff’s alpha ≥ 0.80 indicating high agreement, 0.667–0.79 indicating moderate agreement, and < 0.667 indicating poor agreement [24]. After assessing normal distribution of the data, mixed models fitted with the restricted maximum likelihood method, REML [25], were used to analyze the effect of acceleration level (2D: 2.3, 4, 6, and 8; 3D: 8, 10, and 13) and reconstruction method (CS *versus* CS-AI) on indicators of subjective (overall impression, artifacts, and delineation of the subscapularis tendon, the bone, the acromioclavicular joint, and the glenoid labrum) and objective image quality (signal- and contrast-to-noise-ratio as well as the structural similarity index measure). As post hoc tests Sidak’s multiple comparisons test [26] was used to compare the different reconstruction methods (CS *versus* CS-AI) at the different acceleration levels. Additionally, Dunnett’s multiple comparisons test [27] was used to compare all sequences to the reference sequences (2D: CS 2.3; 3D: CS 8). All post hoc tests were corrected for multiple comparisons. Data are reported as the mean ± SD. A *p* value below 0.05 was considered statistically significant. A priori sample size calculation was performed using G*power 3.1.9.7 based on previous results for acceleration techniques in knee imaging [12]. A minimum number of 19 volunteers are needed to detect a difference of 0.2 points on the Likert scale with 0.3 SD, alpha = 0.05, and a power of 0.8.

## Results

### Study population

Twenty young, healthy volunteers were included. They were 9 males and 11 females; age 30.75 ± 4.45 years, range 23 − 37 years; weight 69.95 ± 9.40 kg, range 53 − 91 kg; and height 172.60 ± 9.00 cm, range 160 − 186 cm).

### Image analysis

**Fig. 1:**
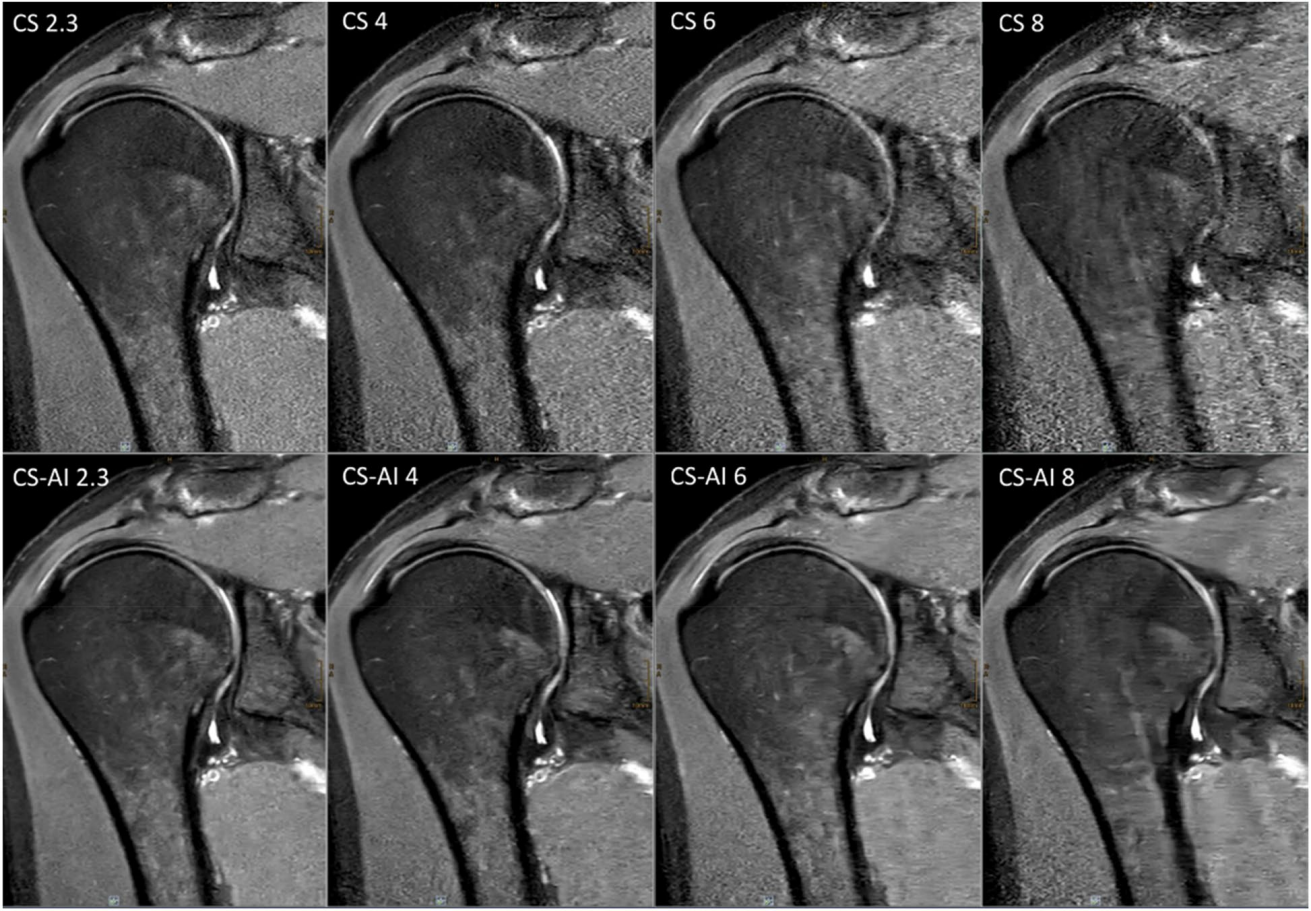
Comparison of a two-dimensional sequence reconstructed using conventional compressed sensing (CS) and compressed sensing combined with a deep learning-based algorithm (CS-AI) with acceleration levels of 2.3, 4, 6, and 8. Note the decreasing image quality with increasing acceleration factor, as well as the enhanced image quality of the CS-AI images compared to the equivalent CS images

**Fig. 2:**
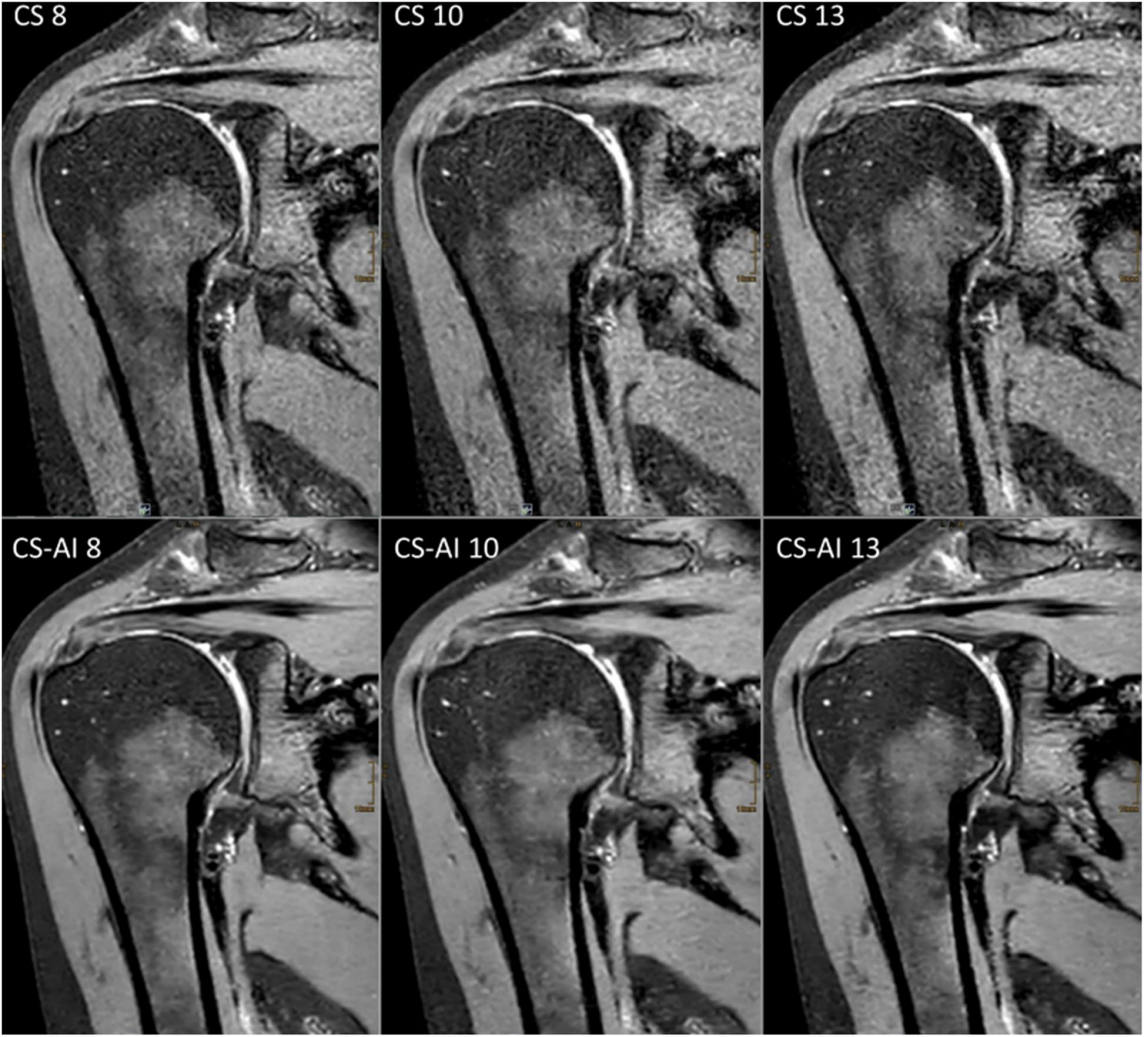
Comparison of a three-dimensional sequence reconstructed conventional compressed sensing (CS) and compressed sensing combined with a deep learning-based algorithm (CS-AI) with acceleration levels of 8, 10, and 13. Note the enhanced image quality of the CS-AI images compared to the equivalent CS images, especially the reduced image noise and the clear delineation of the glenoid labrum

**Fig. 3:**
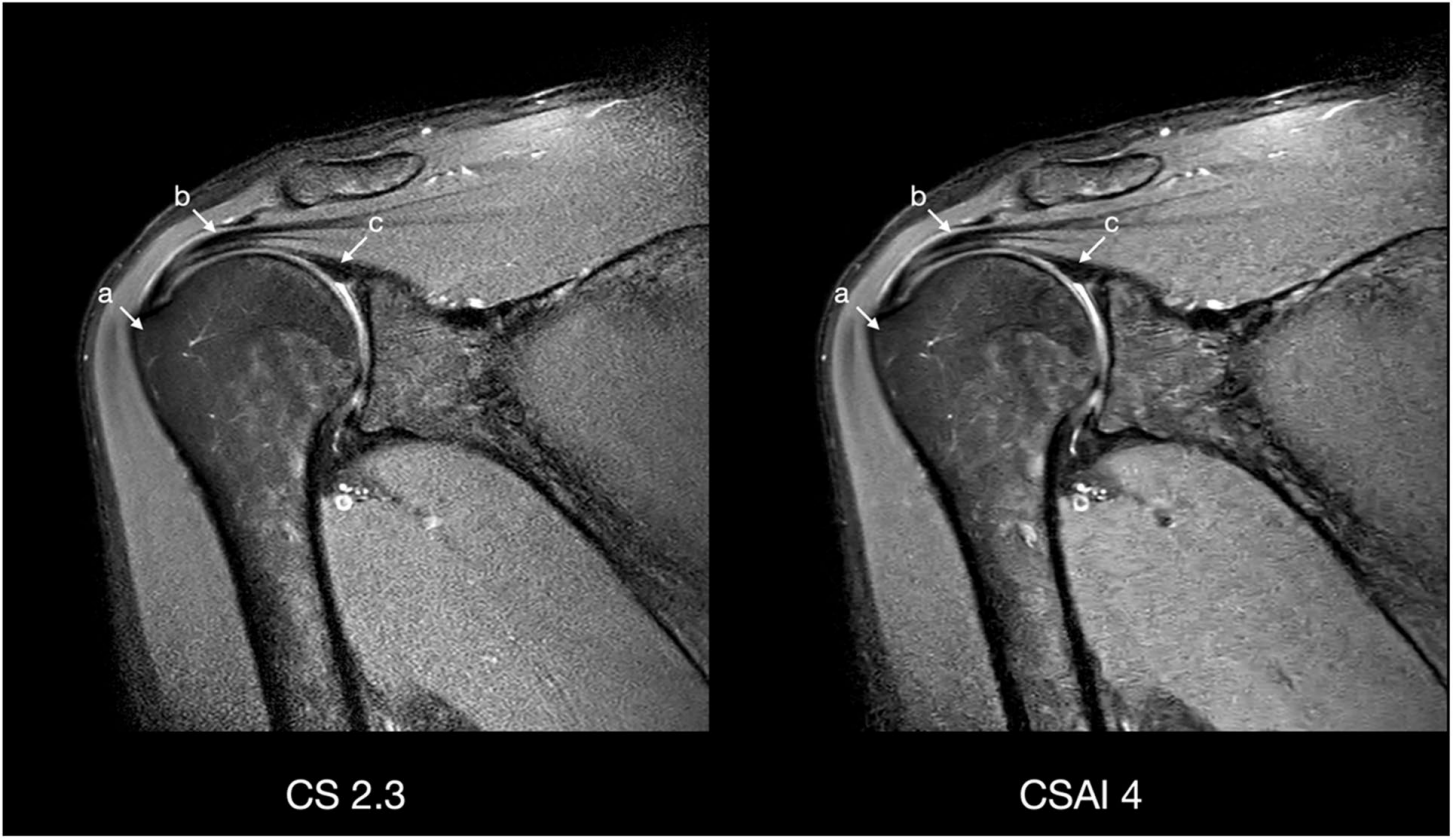
Comparison of a two-dimensional sequence reconstructed using conventional compressed sensing (CS) with acceleration factor 2.3 and compressed sensing combined with a deep learning-based algorithm (CS-AI) with acceleration factor 4 in coronal plane illustrating clear delineation of the greater tuberosity (a), supraspinatus tendon (b), and biceps anchor (c)

**Fig. 4:**
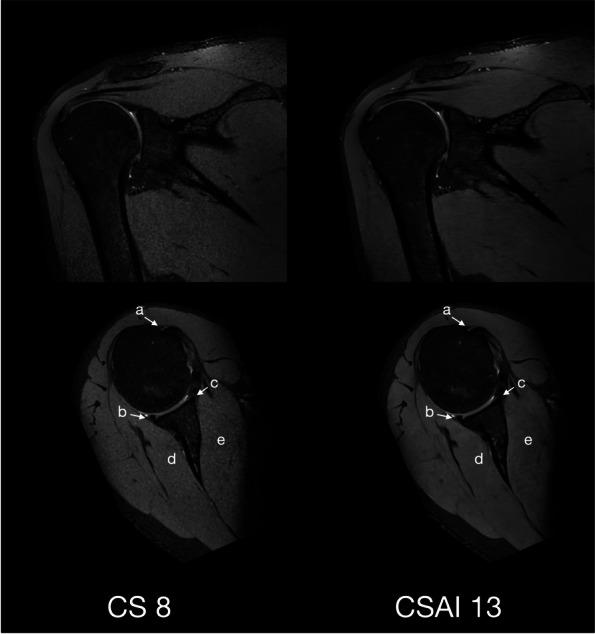
Comparison of a three-dimensional sequence reconstructed using conventional compressed sensing (CS) with acceleration factor 8 and compressed sensing combined with a deep learning-based algorithm (CS-AI) with acceleration factor 13 in coronal and transverse plane illustrating clear delineation of the biceps tendon (a), posterior labrum (b), anterior labrum (c), infraspinatus muscle (d), and subscapular muscle (e)

**Fig. 5:**
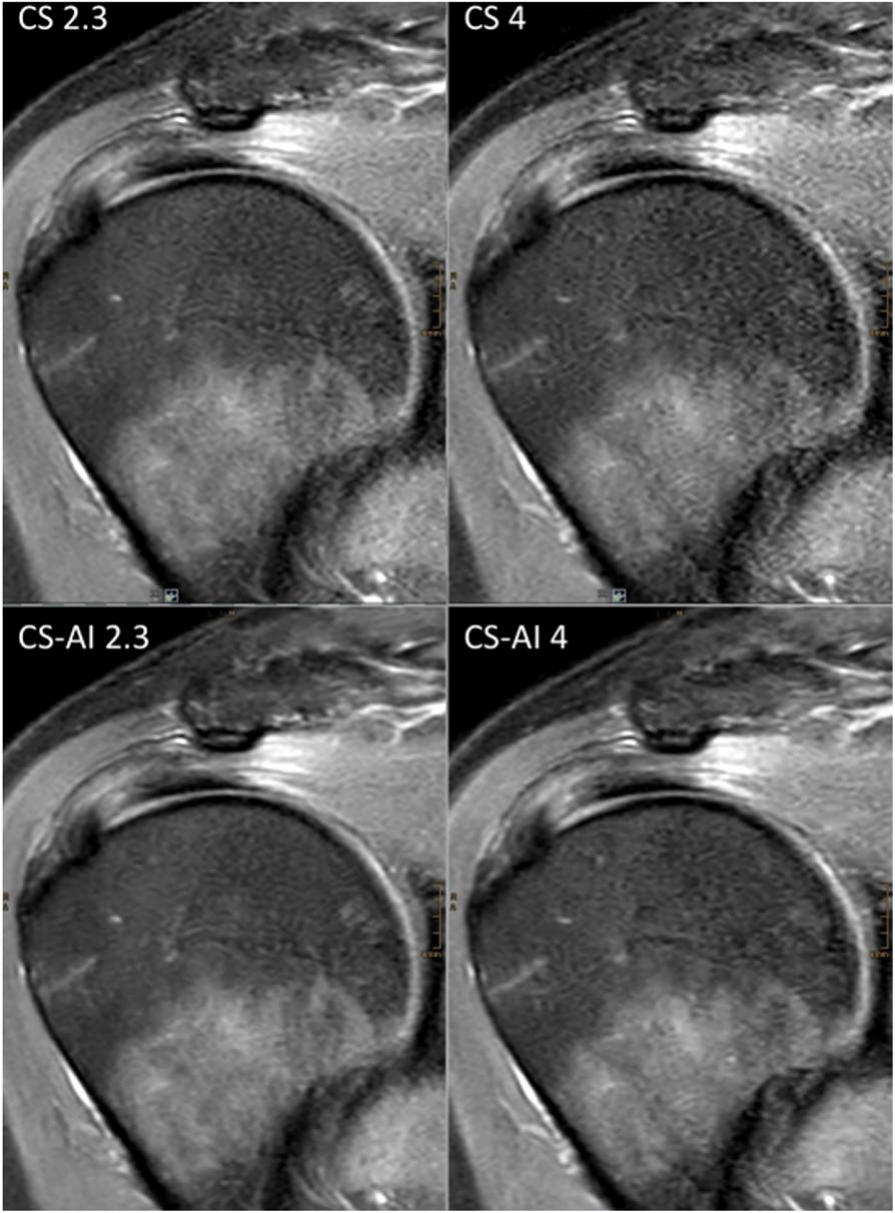
Comparison of a two-dimensional sequence reconstructed using conventional compressed sensing (CS) and compressed sensing combined with a deep learning-based algorithm (CS-AI) with acceleration levels 2.3 and 4, showing mild insertional tendinopathy of the supraspinatus tendon

Scan time decreased with increasing CS factor for both 2D and 3D sequences. An overview of the duration of the sequences is shown in Table 1. Figures 1 and 2 show reconstructions of a 2D and 3D sequence using CS and CS-AI at the respective acceleration levels. Figures 3 and 4 illustrate clear delineation of anatomical landmarks for 2D and 3D sequences. Upon review of the acquired images, it was discovered that one participant suffered from mild insertional tendinopathy of the M. supraspinatus tendon. Figure 5 shows the pathology in a 2D sequence with acceleration factors 2.3 and 4 reconstructed using CS and CS-AI.

### Subjective image analysis

Interrater agreement was assessed using Krippendorff´s alpha, indicating moderate interrater agreement for the subjective scoring over all acceleration factors (Krippendorff’s alpha = 0.72). Mixed models (restricted maximum likelihood method) demonstrated a significant effect of the acceleration factors on the subjective measures of image quality (*p* < 0.001).

**Fig. 6:**
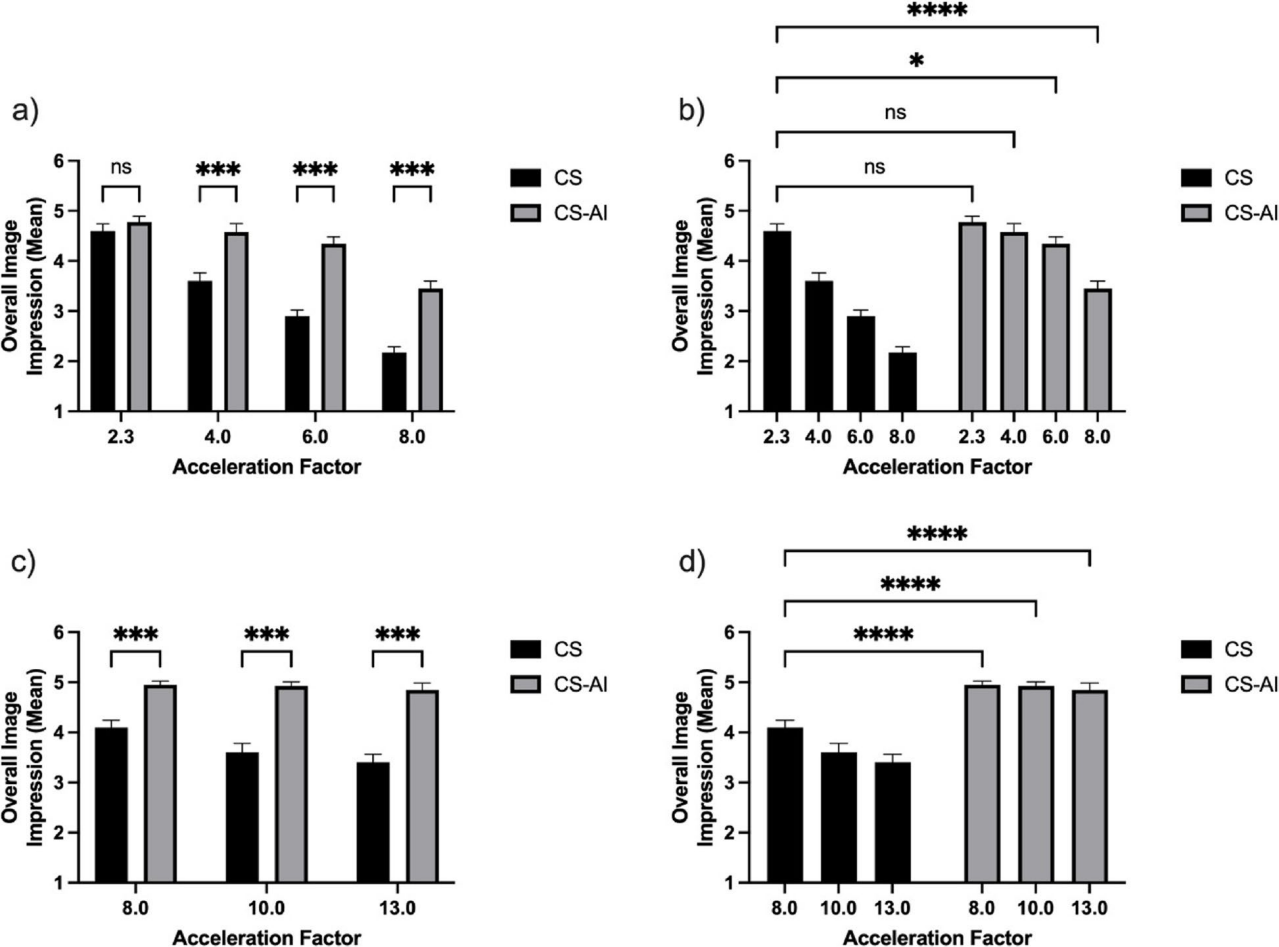
Mean subjective ratings of the overall image quality for two-dimensional (**a**, **b**) and three-dimensional (**c**, **d**) sequences reconstructed using compressed sensing (CS) and compressed sensing combined with a deep learning-based algorithm (CS-AI). The comparison to the respective reference sequences is shown in **b** and **d**. *p ≤ 0.026, **p < 0.010, ***p < 0.001, and ****p < 0.0001

Images reconstructed using CS-AI were rated significantly better than the respective sequences reconstructed using CS for all acceleration levels and all evaluated criteria (all *p* ≤ 0.011; see Table 3), except for the delineation of the bone, the glenoid labrum, and the overall image impression in the 2D sequences with the acceleration factor 2.3, where there was no significant difference between CS and CS-AI sequences; Fig. 6 shows the mean subjective ratings of the overall image quality.

**Table 3:** Mean values and standard deviation for the subjective reading

	Two-dimensional sequences				Three-dimensional sequences		
	CS 2.3 CS-AI 2.3	CS 4 CS-AI 4	CS 6 CS-AI 6	CS 8 CS-AI 8	CS 8 CS-AI 8	CS 10 CS-AI 10	CS 13 CS-AI 13
**Subscapularis tendon**							
**CS**	4.68 ± 0.29 /*	3.83 ± 0.24 */*	3.20 ± 0.47 */*	2.38 ± 0.39 */*	4.05 ± 0.22 /*	4.00 ± 0.16 -/*	3.93 ± 0.29 -/*
**CS-AI**	4.98 ± 0.11 */*	4.83 ± 0.37 -/*	4.87 ± 0.23 -/*	3.58 ± 0.47 */*	5.00 ± 0.00 */*	5.00 ± 0.00 */*	5.00 ± 0.00 */*
**Bone**							
**CS**	4.95 ± 0.15 /-	4.00 ± 0.28 */*	3.80 ± 0.30 */*	3.33 ± 0.37 */*	4.28 ± 0.26 /*	4.15 ± 0.24 -/*	4.03 ± 0.20 */*
**CS-AI**	5.00 ± 0.00 -/-	4.95 ± 0.15 -/*	4.97 ± 0.11 -/*	4.48 ± 0.20 */*	5.00 ± 0.00 */*	5.00 ± 0.00 */*	5.00 ± 0.00 */*
**Acromioclavicular joint**							
**CS**	4.63 ± 0.28 /*	3.60 ± 0.38 */*	3.05 ± 0.28 */*	2.50 ± 0.36 */*	3.90 ± 0.21 /*	3.68 ± 0.34 */*	3.40 ± 0.35 */*
**CS-AI**	4.90 ± 0.21 */*	4.75 ± 0.38 -/*	4.61 ± 0.32 -/*	3.73 ± 0.44 */*	4.98 ± 0.11 */*	4.95 ± 0.15 */*	4.90 ± 0.21 */*
**Glenoid labrum**							
**CS**	4.53 ± 0.34 /-	3.35 ± 0.40 */*	2.60 ± 0.50 */*	1.98 ± 0.41 */*	3.70 ± 0.30 /*	3.15 ± 0.33 */*	3.08 ± 0.37 */*
**CS-AI**	4.83 ± 0.29 -/-	4.50 ± 0.54 -/*	4.50 ± 0.41 -/*	3.30 ± 0.55 */*	4.48 ± 0.20 */*	4.43 ± 0.18 */*	4.25 ± 0.30 */*
**Artifacts**							
**CS**	4.33 ± 0.29 /*	3.33 ± 0.37 */*	2.40 ± 0.45 */*	1.63 ± 0.28 */*	3.80 ± 0.30 /*	3.23 ± 0.30 */*	3.25 ± 0.26 */*
**CS-AI**	4.83 ± 0.34 */*	4.40 ± 0.48 -/*	4.24 ± 0.35 -/*	3.15 ± 0.52 */*	4.93 ± 0.18 */*	4.78 ± 0.30 */*	4.65 ± 0.24 */*
**Overall image impression**							
**CS**	4.60 ± 0.31 /-	3.60 ± 0.35 */*	2.90 ± 0.26 */*	2.18 ± 0.24 */*	4.10 ± 0.31 /*	3.60 ± 0.38 */*	3.40 ± 0.35 */*
**CS-AI**	4.78 ± 0.26 -/-	4.58 ± 0.37 -/*	4.34 ± 0.29 */*	3.45 ± 0.32 */*	4.95 ± 0.15 */*	4.93 ± 0.18 */*	4.85 ± 0.29 */*

*CS* Compressed sensing, *CS-AI* Compressed sensing combined with a deep learning-based algorithm

*/* denotes statistically significant differences (*p* ≤ 0.026) compared to the reference sequence (2D CS 2.3 or 3D CS 8.0) (* before diagonal slash) or the corresponding reconstruction with the same acceleration level (CS *versus* CS-AI) (* after diagonal slash)

Regarding the comparison to the 2D reference sequence (CS 2.3), ratings for the sequences reconstructed using CS-AI did not differ significantly from the reference sequence for the delineation of the subscapularis tendon, the bone, the acromioclavicular joint, and the glenoid labrum as well as visible artifacts for an acceleration factor up to 6 and for the overall image quality for an acceleration factor up to 4 (all *p* ≥ 0.221 see Table 3).

Regarding the comparison to the 3D reference sequence (CS 8), superior ratings for the reconstruction using CS-AI were obtained for all criteria of subjective image quality for an acceleration factor up to 13 (all *p* < 0.001).

### Objective image analysis

**Fig. 7:**
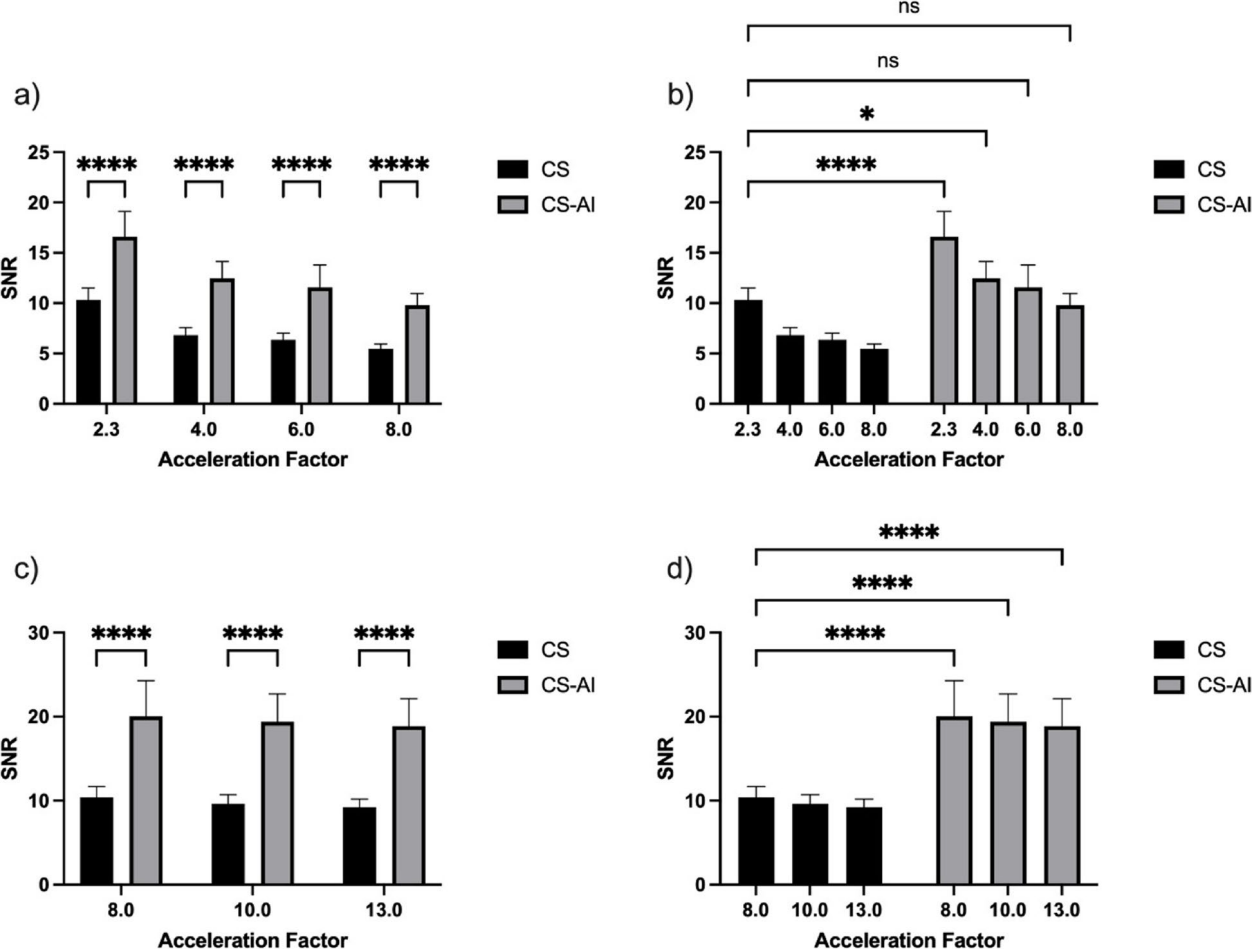
Mean signal-to-noise-ratio for muscle for two-dimensional (**a**, **b**) and three-dimensional (**c**, **d**) sequences reconstructed using compressed sensing (CS) and compressed sensing combined with a deep learning-based algorithm (CS-AI). The comparison to the respective reference sequences is shown in **b** and **d**. * *p* ≤ 0.025, ** *p* < 0.01, *** *p* < 0.001, and **** *p* < 0.0001

Mixed models (restricted maximum likelihood method) demonstrated a significant effect of the acceleration factors on the objective measures of image quality (*p* < 0.001). SNR of the bone and muscle as well as CNR of the bone/muscle and tendon/muscle were significantly higher in the sequences reconstructed using CS-AI compared to the sequences reconstructed using CS for all acceleration levels (all *p* ≤ 0.003; see Table 4); Fig. 7 shows the mean signal-to-noise-ratio for muscle. There was a slight tendency for SNR of the bone to increase with higher acceleration levels in the 3D sequences; however, this increase was not statistically significant. SNR tendon did not differ significantly between both reconstruction algorithms (all *p* ≥ 0.589). CNR bone-tendon was significantly higher for the 2D CS-AI sequences reconstructed with an acceleration factor of 8 (*p* = 0.002) and the 3D CS-AI sequences reconstructed with an acceleration factor of 8 (*p* = 0.006) and 10 (*p* = 0.001; see Table 4).

**Table 4:** Mean values and standard deviation for SNR and CNR

	Two-dimensional sequences				Three-dimensional sequences		
	CS 2.3 CS-AI 2.3	CS 4 CS-AI 4	CS 6 CS-AI 6	CS 8 CS-AI 8	CS 8 CS-AI 8	CS 10 CS-AI 10	CS 13 CS-AI 13
**SNR bone**							
**CS**	8.09 ± 1.50 /*	6.12 ± 1.19 */*	6.82 ± 1.45 */*	5.87 ± 1.25 */*	4.29 ± 0.78 /*	4.33 ± 0.80 -/*	4.59 ± 1.16 */*
**CS-AI**	9.25 ± 2.14 */*	7.03 ± 1.56 */*	8.01 ± 1.76 -/*	7.94 ± 2.00 -/*	5.34 ± 1.28 */*	5.67 ± 1.51 */*	5.83 ± 1.26 */*
**SNR muscle**							
**CS**	10.31 ± 2.57 /*	6.81 ± 1.61 */*	6.36 ± 1.44 */*	5.44 ± 1.06 */*	10.40 ± 2.77 /*	9.61 ± 2.38 -/*	9.21 ± 2.08 -/*
**CS-AI**	16.60 ± 5.36 */*	12.47 ± 3.55 */*	11.58 ± 4.61 -/*	9.80 ± 2.49 -/*	20.03 ± 9.09 */*	19.39 ± 7.08 */*	18.85 ± 7.01 */*
**SNR tendon**							
**CS**	3.21 ± 1.66 /-	2.57 ± 1.09 */-	2.77 ± 1.00 -/-	2.71 ± 1.14 -/-	3.80 ± 1.60 /-	3.90 ± 2.35 -/-	3.56 ± 1.90 -/-
**CS-AI**	3.17 ± 1.67 -/-	2.52 ± 1.27 */-	2.90 ± 1.32 -/-	2.84 ± 1.76 -/-	3.81 ± 1.60 -/-	4.05 ± 2.66 -/-	3.42 ± 1.66 -/-
**CNR bone/tendon**							
**CS**	1.16 ± 0.92 /-	0.92 ± 0.59 -/-	1.06 ± 0.77 -/-	0.81 ± 0.54 */*	1.09 ± 0.83 /*	1.14 ± 1.03 -/*	0.93 ± 0.74 */-
**CS-AI**	1.27 ± 1.13 -/-	1.03 ± 0.72 -/-	1.20 ± 0.98 -/-	1.00 ± 0.78 -/*	1.22 ± 0.86 */*	1.32 ± 1.22 */*	1.03 ± 0.83 -/-
**CNR tendon/muscle**							
**CS**	3.89 ± 1.97 /*	2.99 ± 1.24 */*	2.90 ± 1.29 */*	2.49 ± 0.86 */*	3.27 ± 1.28 /*	3.21 ± 1.55 -/*	3.24 ± 1.24 -/*
**CS-AI**	4.68 ± 2.86 */*	3.89 ± 1.90 -/*	3.89 ± 2.56 -/*	3.50 ± 1.57 -/*	3.92 ± 1.70 */*	4.13 ± 2.25 */*	3.99 ± 1.93 */*
**CNR bone/muscle**							
**CS**	4.21 ± 1.54 /*	2.92 ± 0.95 */*	2.72 ± 0.96 */*	2.29 ± 0.78 */*	5.28 ± 1.21 /*	4.83 ± 1.21 -/*	4.80 ± 1.24 -/*
**CS-AI**	5.98 ± 2.59 */*	4.57 ± 1.71 -/*	4.14 ± 1.92 -/*	3.89 ± 1.60 -/*	7.79 ± 2.29 */*	7.75 ± 2.70 */*	7.82 ± 2.60 */*
**SSIM**							
**CS**	1.00 ± 0.00 /*	0.57 ± 0.10 */*	0.56 ± 0.07 */*	0.50 ± 0.08 */*	1.00 ± 0.00 /*	0.64 ± 0.08 */*	0.63 ± 0.08 */*
**CS-AI**	0.92 ± 0.03 */*	0.64 ± 0.11 */*	0.65 ± 0.08 */*	0.62 ± 0.08 */*	0.86 ± 0.03 */*	0.68 ± 0.09 */*	0.69 ± 0.09 */*

*CS* compressed sensing, *CS-AI* compressed sensing combined with a deep learning-based algorithm, *CNR* contrast-to-noise ratio, *SNR* signal-to-noise ratio, *SSIM* structural similarity index measure

*/* marking significant difference (*p* ≤ 0.025) compared to the reference sequence (* before diagonal slash) or the corresponding reconstruction with the same acceleration level (* after diagonal slash)

Regarding the comparison to the 2D reference sequence (CS 2.3), sequences reconstructed using CS-AI did not differ significantly from the reference sequence with regard to the SNR bone, muscle and tendon as well as CNR bone-muscle, tendon–muscle, and bone-tendon for an acceleration factor up to 8 (all *p* ≥ 0.395).

Regarding the comparison to the 3D reference sequence (CS 8), sequences reconstructed using CS-AI did not differ significantly from the reference sequence with regard to the SNR tendon and CNR bone-tendon for an acceleration factor up to 13 (all *p* ≥ 0.058) and scored significantly higher with regard to SNR bone and muscle as well as CNR tendon–muscle and CNR bone-muscle than the reference sequence for an acceleration factor up to 13 (all *p* ≤ 0.001).

As for the structural similarity index measure, images reconstructed CS-AI received significantly higher values compared to the sequences reconstructed using CS for all acceleration levels (all *p* ≤ 0.001; see Table 4).

## Discussion

The aim of the current study was to compare the subjective and objective image quality of a conventional newly developed image reconstruction algorithm for compressed sensing at different acceleration factors for 2D and 3D imaging of the shoulder. Images reconstructed using CS-AI scored on average significantly higher on both subjective and objective measures of image quality for 2D and 3D compared to the respective images reconstructed using CS. These results are in line with previous studies that have shown similar performance using the same algorithm. For instance, Fervers et al. [28] showed significantly better subjective image quality for 3D T2-weighted images of the lumbar spine reconstructed using CS-AI compared to images reconstructed using CS. Additionally, higher objective and subjective image quality for sequences reconstructed using CS-AI have been reported for ankle and prostate [29, 30].

However, even though CS-AI does produce images with a higher subjective and objective image quality compared to CS, the images may still not be of diagnostic quality if the acceleration level is too high. Therefore, we compared the images generated using CS-AI at the different acceleration levels to the reference sequence used in current clinical practice (2D CS 2.3; 3D CS 8) to find an optimal acceleration factor that generates images with similar subjective and objective image quality than the current clinical standard. Considering both objective and subjective image quality, 2D sequences reconstructed using CS-AI with a 4-fold acceleration did not perform significantly worse than the same sequences reconstructed using regular CS with a 2.3-fold acceleration, and 3D sequences reconstructed using CS-AI with a 13-fold acceleration did not perform significantly worse than the same sequences reconstructed using regular CS with an 8-fold acceleration.

Translating these acceleration factors into acquisition times, replacing the standard CS 2.3 sequence with the CS-AI 4 sequence (167 s *versus* 100 s) would result in 40% less acquisition time for 2D sequences and 38% for 3D sequences (standard CS 8 sequence with 289 *versus* CS-AI 13 with 179 s). Figures 3 and 4 illustrate clear delineation of anatomical landmarks at these two acceleration factors compared to the reference sequences (2D: CS 2.3 *versus* CS-AI 4; 3D: CS 8 *versus* CS-AI 13). Faster image acquisition can improve the efficiency of an imaging center and increase patient access to imaging. Additionally, decreasing the time patients spend in the scanner can help reduce motion artifacts, thereby increasing image quality and diagnostic accuracy, and improving overall patient comfort.

However, this study has several limitations. First, our study only included a small sample size of healthy volunteers. Even though the results of the two readers show that the delineation of anatomical structures was not inferior to the reference sequences, as a next step, patients with common shoulder pathologies should be scanned to ensure that image quality of pathological findings is also preserved. One of the common arguments against DL-based reconstruction algorithms is the fear of losing information, whereby pathological findings are replaced by normal anatomy from the training data. To counteract this, the multiscale network used in this study includes the integration of a data consistency term per coil element comparing the reconstructed data with the originally acquired data to ensure consistency [17]. Both objective and subjective image quality for sequences reconstructed using CS-AI was generally higher compared to conventional CS, showing that, based on the parameters measured in this study, there was no evidence for loss of information. Additionally, studies including patients with pathologies using the same CS-AI algorithm as in our study found no evidence for loss of information. For instance, Bischof et al. [30] found no difference in PI-RADS scores between images reconstructed using CS-AI compared to images reconstructed using CS. Additionally, Feuerriegel et al. [31] found no loss of diagnostic information for common shoulder pathologies in images acquired with an acceleration factor of 2.5. As for other reconstruction algorithms of the shoulder, both Hahn et al. [32] and Kaniewska [33] et al. showed similar or better delineation of common shoulder pathologies using a deep learning based reconstruction technique to reduce motion artifacts based on the PROPELLER method, providing further evidence that DL-based techniques may accurately reproduce pathologies and not lead to loss of information. Nevertheless, future studies should include more participants as well as patients with different pathologies to further evaluate the accuracy of the algorithm.

Second, our study only focused on a 2D and 3D proton density-weighted sequence of the shoulder. Whereas studies have shown similar performance of DL-based reconstruction algorithms across different MRI sequences [34], there are also studies showing that performance can differ between MRI sequences [35]. Thus, future studies should also include other sequences besides proton density sequences (*e.g.*, T1- and T2-weighted sequences) to ensure that objective and subjective image quality are equally well preserved in a wider range of sequences.

Third, our study did not include a reference sequence without acceleration. The clinically used sequences in our institution (a sequence with a 2.3-fold acceleration for 2D images and an 8-fold acceleration for 3D images) was used as the respective reference standard. Choosing accelerated sequences as the reference standard may pose the risk of using a reference standard that already has reduced image quality. Showing non-inferiority against this reference standard may hide the fact that the image quality of the accelerated images is lower than the standard sequence without acceleration. Continually establishing faster reference standards and testing only for non-inferiority against the prior reference standard may pose the risk of hiding a continuous decrease in image quality. Thus, besides testing for non-inferiority, measures of image quality should also be interpreted with regard to an absolute standard. In our study, the non-inferior accelerated 2D sequence received an average rating of 4.58 and the 3D sequence of 4.85 from both readers. In the context of the 5-point rating scale used in our study, these scores lie in the middle between a score of 4 (good for majority of diagnoses) to 5 (optimal). Therefore, even though we did not use a reference standard without acceleration, based on the results of the two readers, the accelerated sequences are of sufficient diagnostic quality.

In conclusion, the results of our study show that the combination of deep-learning and compressed sensing hold the potential for further scan time reduction in 2D and 3D imaging of the shoulder while providing overall better objective and subjective image quality compared to the conventional approach. The implementation of this algorithm can help increase patient access to imaging and reduce motion artefacts by decreasing the overall time patients spend in the scanner. The results encourage further clinical investigation, extending the use cases to a clinical population and a wider range of MRI sequences.

## Data Availability

The data underlying this article cannot be shared publicly for the privacy of individuals that participated in the study. The data will be shared on reasonable request to the corresponding author.

## Abbreviations

2D: Two-dimensional
3D: Three-dimensional
AI: Artificial intelligence
CNR: Contrast-to-noise ratio
CS: Compressed sensing
DL: Deep learning
ROI: Region of interest
SD: Standard deviation
SENSE: Sensitivity encoding
SNR: Signal-to-noise ratio
SSIM: Structural similarity index measure
